# Physico-Chemical, In Vitro, and In Vivo Evaluation of a 3D Unidirectional Porous Hydroxyapatite Scaffold for Bone Regeneration

**DOI:** 10.3390/ma10010033

**Published:** 2017-01-03

**Authors:** Manabu Tanaka, Hisao Haniu, Takayuki Kamanaka, Takashi Takizawa, Atsushi Sobajima, Kazushige Yoshida, Kaoru Aoki, Masanori Okamoto, Hiroyuki Kato, Naoto Saito

**Affiliations:** 1 Department of Orthopaedic Surgery, School of Medicine, Shinshu University, Asahi 3-1-1, Matsumoto, Nagano 390-8621, Japan; hhaniu@shinshu-u.ac.jp (H.H.); kam17@hotmail.co.jp (T.K.); takashitak@shinshu-u.ac.jp (T.T.); soba@shinshu-u.ac.jp (A.S.); gooddays83@yahoo.co.jp (K.Y.); kin29men@shinshu-u.ac.jp (K.A.); masanori_ckmt@ybb.ne.jp (M.O.); hirokato@shinshu-u.ac.jp (H.K.); 2 Institute for Biomedical Sciences, Shinshu University, Asahi 3-1-1, Matsumoto, Nagano 390-8621, Japan; saitoko@shinshu-u.ac.jp

**Keywords:** regenerative medicine, scaffold, unidirectional pore

## Abstract

The unidirectional porous hydroxyapatite HAp (UDPHAp) is a scaffold with continuous communicated pore structure in the axial direction. We evaluated and compared the ability of the UDPHAp as a three-dimensional (3D) bone tissue engineering scaffold to the interconnected calcium porous HAp ceramic (IP-CHA). To achieve this, we evaluated in vitro the compressive strength, controlled rhBMP-2 release behavior, adherent cell morphology, cell adhesion manner, and cell attachment of UDPHAp. As a further in vivo experiment, UDPHAp and IP-CHA with rhBMP-2 were transplanted into mouse calvarial defects to evaluate their bone-forming ability. The Results demonstrated that the maximum compressive strengths of the UDPHAp was 7.89 ± 1.23 MPa and higher than that of IP-CHA (1.92 ± 0.53 MPa) (*p* = 0.0039). However, the breaking energies were similar (8.99 ± 2.72 vs. 13.95 ± 5.69 mJ, *p* = 0.055). The UDPHAp released rhBMP-2 more gradually in vivo. Cells on the UDPHAp adhered tightly to the surface, which had grown deeply into the scaffolds. A significant increase in cell number on the UDPHAp was observed compared to the IP-CHA on day 8 (102,479 ± 34,391 vs. 32,372 ± 29,061 estimated cells per scaffold, *p* = 0.0495). In a mouse calvarial defect model, the percentages of new bone area (mature bone + trabecular bone) in the 2x field were 2.514% ± 1.224% for the IP-CHA group and 7.045% ± 2.055% for the UDPHAp group, and the percentage was significantly higher in the UDPHAp group (*p* = 0.0209). While maintaining the same strength as the IP-CHA, the UDPHAp with 84% porosity showed a high cell number, high cell invasiveness, and excellent bone formation. We believe the UDPHAp is an excellent material that can be applied to bone regenerative medicine.

## 1. Introduction

For the treatment of large bone defects induced by fracture or tumor resection, the best way to regenerate bone in defect reconstruction is the use of autologous bone graft, but there are several drawbacks, including the amount of available bone and pain at the donor site [1]. Allograft is also performed, which has problems including infection and immunoreaction [1]. For these reasons, clinical applications of artificial bone as a substitute for those grafts has been developed rapidly [2]. Synthetic biomaterials [2] (bioabsorbable hydrogels, polylactic acid, hydroxyapatite (HAp), beta tricalcium-phosphates, etc.) are currently being utilized as scaffolds for bone tissue engineering [3]. In the field of bone tissue engineering, scaffolds of various materials with various 3D structures have been developed to build 3D structures of cells and enable the controlled release of drugs [4]. HAp [5,6,7] is broadly used, as well as bioabsorbable polymer [8]. HAp is used as an artificial bone to make up for bone defect sites due to its strength, bone conduction ability, and their similarity to the mineral component of bone [9,10]. Unidirectional porous HAp (UDPHAp) [11] has been developed for orthopedic applications as a bone void filler with 75% porosity and penetrating oval pores that range in diameter from 100 to 300 µm. Interconnected porous calcium HAp (IP-CHA) [12] is a widely available bone substitute that has a fully interconnected porous structure with 75% porosity. IP-CHA has also been studied in the field of bone and joint regeneration and tissue engineering, because interconnected pores are beneficial for culturing cells and retaining growth factors [13]. The use of IP-CHA enables a deep penetration of osseous tissue ingrowth and is widely commercialized. The recent commercialization of scaffolds with unidirectionally connected pores for tissue regeneration has been gaining attention in recent years [6,7]. In this study, we highlighted the use of UDPHAp as a 3D scaffold, developed for the purpose of bone tissue engineering. The UDPHAp exhibits high liquid permeation, tissue penetration, and bone conduction ability [11]. Iwasashi et al. reports the clinical application of UDPHAp as an artificial bone alternative for a bone defect site [14]. Previous works describe the results of in vivo experiments. To our knowledge, no study discusses the in vivo bone regenerative effect of UDPHAp in conjunction with in vitro experiments. This is the first report to compare the clinical merit of the UDPHAp and IP-CHA through both in vivo and in vitro experiments. Scaffold materials require surface suitability for cell growth, strong coupling of the scaffold material with bone matrix, sufficient strength of the material to provide mechanical stability in the field of tissue engineering, and penetration of tissues and blood vessels [15]. We evaluated the in vitro and in vivo capacity of the UDPHAp as a 3D scaffold for bone regenerative medicine in comparison to the IP-CHA using UDPHAp with 84% porosity, a scaffold developed for tissue engineering and 3D cell culture.

## 2. Materials and Methods

### 2.1. Ethics Statement

All experiments were carried out according to institutional guidelines for animal experimentation at Shinshu University School of Medicine. All protocols used in this study were reviewed and approved by the Division of Laboratory Animal Research (#260021). All surgery was performed under general anesthesia (intraperitoneal injection of sodium pentobarbital), and all efforts were made to minimize suffering. All mice and rats were euthanized using isoflurane inhalation at the end of the study.

### 2.2. Scaffolds

UDPHAp (Kurarife; Kuraray Medical, Inc., Tokyo, Japan) is fabricated by promoting unidirectional ice column growth in an aqueous suspension of HAp granules. UDPHAp was developed by unidirectionally cooling the HAp slurry using a cold plate and liquid nitrogen to grow ice columns in a solution of HAp granules. After repeated freeze-drying and thawing, the ice columns formed in the HAp slurry were removed, and capillary pores with a uniform orientation were formed [16]. As a clinical control, we used a fully interconnected porous calcium hydroxyapatite (IP-CHA, NEOBONE, Covalent Materials Co., Tokyo, Japan) [12]. In this study, we used test samples of the UDPHAps and IP-CHAs that were cut into disks of 5 mm in diameter and 2 mm in height.

### 2.3. Scaffold Characterization by Micro-CT before Cell Seeding

Micro-CT has already been established as a standard method for scaffold characterization [17]. We scanned the UDPHAps and IP-CHAs (*n* = 6, respectively) using a micro-CT system (R_mCT; Rigaku Corp., Tokyo, Japan) operated at 40 kV tube voltage, 250 µA tube current, and exposure time of 18 s. Each image was reconstructed by i-View software (J. Morita MFG. Corp., Kyoto, Japan), rendered to 3D by VG Studio MAX software (Volume Graphics GmbH, Heidelberg, Germany), and subsequently evaluated for their microporosity. Threshold value was determined when the background was excluded and the solid structure of the material was all selected (grayscale value 140/255).

### 2.4. Calculation of Porosity and SSA

Three ROIs (regions of interest) were randomly selected for each cylindrical UDPHAp and IP-CHA (diameter, 100 mm; height, 70 mm). To avoid artifacts on edges, ROIs were set around the center of each scaffold. Total volume (TV), scaffold volume (SV), scaffold volume ratio (SV/TV), and surface area (SA) were measured for each scaffold material. Values of porosity and SSA were defined as follows:

(1) $$\text{Porosity}\left(\%\right)=\left(1-\frac{\text{SV}}{\text{TV}}\right)\cdot 100$$

(2) $$\text{SSA}\;\left({\text{mm}}^{2}/{\text{mm}}^{3}\right)=\text{SA}/\text{SV}$$

### 2.5. Strength Test

Uniaxial compression tests were carried out on both the UDPHAp and IP-CHA (*n* = 6). Compressive and tensile strength were loaded against the bottom surface of the cylindrical scaffolds in a perpendicular direction (parallel to the orientation for the UDPHAp) by the Autograph AGS-H (Shimadzu Co, Ltd., Kyoto, Japan) at a compression speed of 1 mm/min.

### 2.6. In Vitro rhBMP-2 Releasing Assay

5 μL of 1 mg/mL rhBMP-2 (ATGen, Seongnam, Korea) solution was dropped (5 μg/sample) onto UDPHAps and IP-CHAs (*n* = 5), dried, and then immersed in 500 µL of sterilized water. Supernatants were retrieved on day 1, 2, 3, 4, 5, 6, and 7. Subsequently, 500 µL of new sterilized water was added. Protein concentration of each supernatant was analyzed by a Micro BCA Protein Assay Kit (Thermo Fisher Scientific Inc., Waltham, MA, USA). 

### 2.7. Cell Culture and Seeding Cells onto the Scaffold

MC3T3-E1 preosteoblasts [18] (RIKEN cell bank, Tsukuba, Japan) were cultured in regular culture media consisting of alpha-modified minimum essential medium (alpha-MEM; Nacalai Tesque, Kyoto, Japan), supplemented with 10% heat-inactivated fetal bovine serum (Biowest, Nuaillé, France) and 1% antibiotic-antimycotic mixed stock solution (Nacalai Tesque, Kyoto, Japan) in a humidified atmosphere of 5% CO_2_ at 37 °C. Cells up to passage 20 were used. Before the experiments, cells were trypsinized using 0.25% trypsin/EDTA (Sigma, St. Louis, MO, USA), seeded onto scaffolds in 96-well plates at a density of 1 × 10^4^ cells/scaffold, and suspended in 20 µL of media. They were incubated in a CO_2_ incubator for an hour at 37 °C and 5% CO_2_ saturated humidity until cells adhered to the scaffold. Each scaffold was then transferred to other 96-well plates and cultured by adding 200 µL of alpha-MEM. Media were replaced twice a week.

### 2.8. Observation of Cells Attached to the Scaffolds’ Surface by a Scanning Electron Microscope

Scaffold surfaces before and after adhesion of the cells were evaluated by a scanning electron microscope (SEM). By the method described in the previous section, MC3T3-E1 cells were seeded at 1 × 10^4^ cells/scaffold (*n* = 3), transferred to another 96-well plate, and cultured in 200 µL of alpha-MEM for four days. Scaffolds were then fixed by 2% glutaraldehyde, dried by 50%–100% ethanol, and sputter-coated by osmium. The shape of the surface, distribution of the pore size, and the morphology of the attached cells were observed by Field Emission Scanning Electron Microscopy (JSM-7600F; JEOL, Tokyo, Japan).

### 2.9. Observation of Cell Adhesion by Fluorescence Microscopy

MC3T3-E1 cells that are broadly used for researching cell proliferation and differentiation on the bone tissue engineering scaffold [19] were seeded at 1 × 10^4^ cells/scaffold (*n* = 3), transferred to another 96-well plate, and cultured in 200 µL of alpha-MEM for four days by the same method described in the previous section. Cells were fixed for an hour with 4% paraformaldehyde, treated an hour with 0.1% Triton-X, and stained with FITC-phalloidin (Sigma Aldrich, St. Louis, MO, USA) to show F-actin (red) + bisbenzimide H33342 fluorochrome trihydrochloride (Nacalai Tesque, Kyoto, Japan) and nuclei (blue) for an hour. After washing in PBS (Nacalai Tesque, Kyoto, Japan), they were observed with a fluorescence microscope (BZ-X700; Keyence, Tokyo, Japan). Each scaffold was cut parallel to the axial direction of the cylinder in the middle, and we observed the cell adhesion style at the top and inside of the scaffold.

### 2.10. Seeding Efficiency Analysis

Seeding efficiency analysis was determined as previously described [20]. MC3T3-E1 cells were seeded in a 96-well plate at a density of 1 × 10^4^ cells/scaffold, suspended in 20 µL of media, and left in the scaffolds for 12 h to attach and adapt to each scaffold architecture (*n* = 5). After this period, the scaffolds were removed, and the remaining cells in the wells were trypsinized and counted using an automatic cell counter (TC20; Bio-Rad, Hercules, CA, USA). The seeding efficiency was calculated using the following equation:

$$\text{Seeding efficiency}\left(\%\right)=\frac{\text{cells added to scaffold}-\text{cells inwells}}{\text{cells added to scaffold}}\cdot 100$$

The values reported are the averages for five specimens of each type of scaffold. The contribution of cell proliferation in the well to absolute seeding efficiency values during the 12-h period is very low and can be ignored [20].

### 2.11. Evaluation of Attached Cell Number

To evaluate the effects of the attached cell number, we cultured MC3T3-E1 cells on the UDPHAp and IP-CHA (*n* = 3, respectively). Cells grown on scaffold surfaces were counted after 1, 4, and 8 days using the Alamar blue assay [21]. Alamar blue assay is used to evaluate cell proliferation [22]. Cells were seeded onto the UDPHAp and IP-CHA by the above-described method at a density of 1 × 10^4^ cells/scaffold, placed in a 96-well plate, and cultured in 200 µL of alpha-MEM medium. Alamar blue assay was performed at 1, 4, and 8 days after seeding. We aspirated each well, and after washing in PBS, 10% 100 µL/well of Alamar blue reagent (Invitrogen, Carlsbad, CA, USA) was added and incubated for an hour at 37 °C. Plates were then read by a plate reader (AF2200, Eppendorf, Hamburg, Germany) to measure the fluorescence intensity at the excitation/emission of 530/590 nm. At each measurement, 1 × 10^3^, 1 × 10^4^, and 1 × 10^5^ cells were seeded onto the plate, and the same assay was performed. A calibration curve between the fluorescent intensity and the number of cells was created to which the fluorescence intensity obtained was converted to an estimated cell number.

### 2.12. Healing of Critical-Size Mouse Calvarial Bone Defects in Combination with rhBMP-2

5 µg of 1 mg/mL rhBMP-2 (equivalent to 5 µg of rhBMP-2) was added to the UDPHAp and IP-CHA scaffolds with a direction of orientation parallel to the sagittal plane. The scaffolds were then dried for 24 h in order to prepare rhBMP-2-containing scaffolds. Six-week-old male ddY mice (SLC, Shizuoka, Japan) were anesthetized with intraperitoneal injection of 30 mg/kg sodium pentobarbital (Kyoritsu Seiyaku, Tokyo, Japan) and 1 mL subcutaneous injection of 1% lidocaine (AstraZeneca, Osaka, Japan). Calvarial defects of 5 mm in diameter were made with a trephine bur, which were implanted with rhBMP-2-containing scaffolds. As a control group, 100 µL of 0.05 mg/mL rhBMP-2 (corresponding to 5 µg of rhBMP-2) dissolved in normal saline (Otsuka Pharmaceutical Co., Ltd., Tokyo, Japan) was injected to the bone defect, after which wound closure was performed (Empty group). After three weeks, the mice were euthanized by isoflurane (Abbott Japan, Tokyo, Japan) inhalation. After the heads were dissected, they were fixed in 10% formalin (Wako Pure Chemical Industries, Osaka, Japan), evaluated with µCT, and prepared for histological examination.

The µCT image of a mouse calvaria was collected at 80 kV tube voltage, 80 μA tube current, and scan time of 16 s. Raw data were reconstituted with i-View software and analyzed. After fixation in formalin, the tissues were decalcified in K-CX solution (Falma Co., Tokyo, Japan) for three days. After being embedded in paraffin, they were sliced into sections of 10 µm using a microtome. After being stained with Hematoxylin (Muto Pure Chemicals, Tokyo, Japan) and Eosin (Wako Pure Chemical Industries, Osaka, Japan), sections were observed with an optical microscope (BX50; Olympus, Tokyo, Japan). Sections were also stained with Masson trichrome staining (Muto Pure Chemicals, Tokyo, Japan). Masson trichrome-stained sections were used to define the collagen secreted in the process of bone formation, in which collagen fibers and woven bones are stained blue and mineralized bones are stained red [23]. Using a 2x objective lens, an image was captured to contain the entire bone defect. Then, the area of the new bone occupied in the 2x field was measured using ImageJ software [24].

### 2.13. Statistical Analysis 

Data were expressed as mean and standard deviation. Each statistical analysis was performed using the Mann-Whitney U test and one-way ANOVA, followed by Tukey’s post hoc test. Values of *p* < 0.05 were considered statistically significant.

## 3. Results

### 3.1. Scaffold Characterization by Micro-CT before Cell Seeding

**Figure 1:**
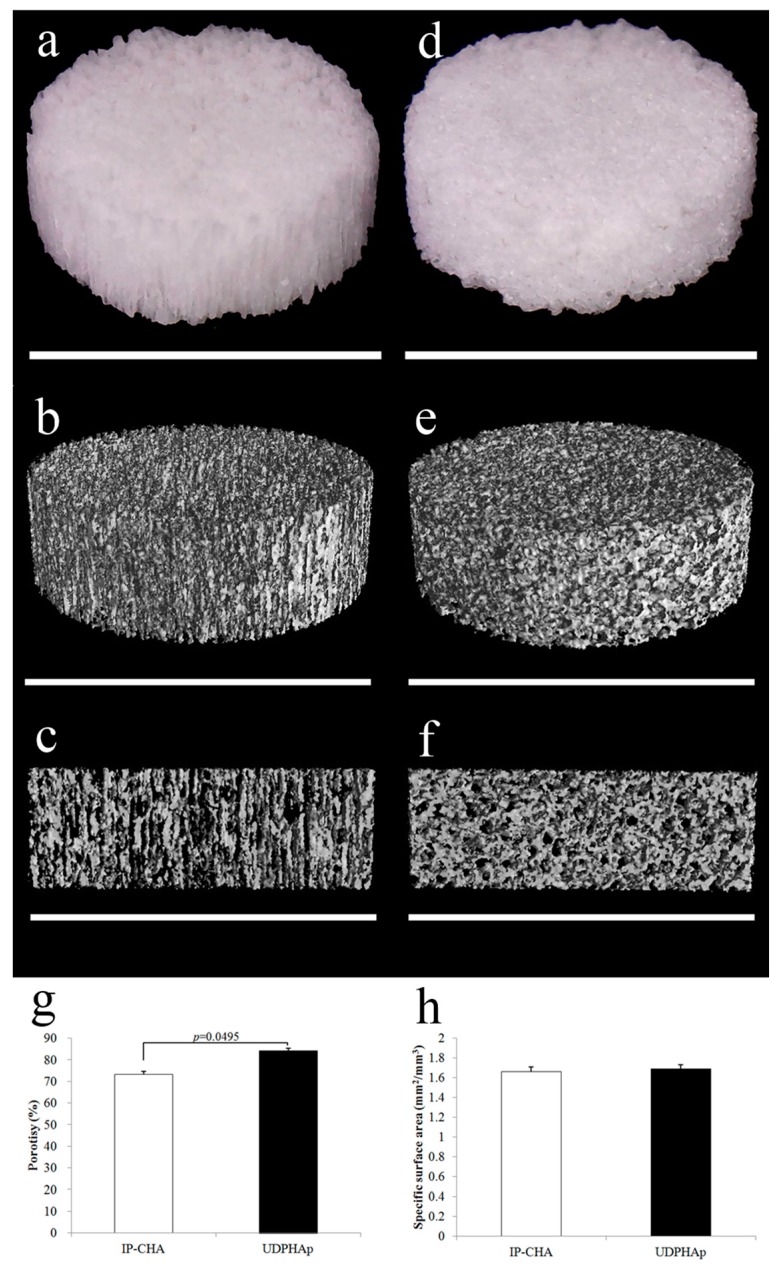
Representative photographs and Micro-CT analysis of porous hydroxyapatite scaffolds. Gross appearance and 3D image renderings from micro-CT scans of (**a**–**c**) UDPHAp and (**b**–**d**) IP-CHA. White bars are 5 mm; (**g**) Porosity and (**h**) SSA of UDPHAp and IP-CHA. Values are mean ± SD; *n* = 3 analyzed by Mann-Whitney U test.

Figure 1 displays the representative photographs and the 3D images of the UDPHAp and IP-CHA scaffolds by micro-CT. Randomly sized spherical macropores were observed in every IP-CHA, and at least one small and identical pore was found as an interconnection within every macropore. On the other hand, the UDPHAp has penetrating oval pores ranging in diameter from 100 to 300 µm, which have interconnected structures. The porosities of the UDPHAp and IP-CHA were 84.2 ± 1.1 and 73.2% ± 1.5%, respectively. Those of the UDPHAp were significantly higher (*p* = 0.0495) than the IP-CHA. In addition, SSAs were 1.69 ± 0.04 and 1.66 ± 0.05 mm^2^/mm^3^, and there was no significant difference between the two groups (*p* = 0.2752) (Figure 1g,h).

### 3.2. Strength Test

**Figure 2:**
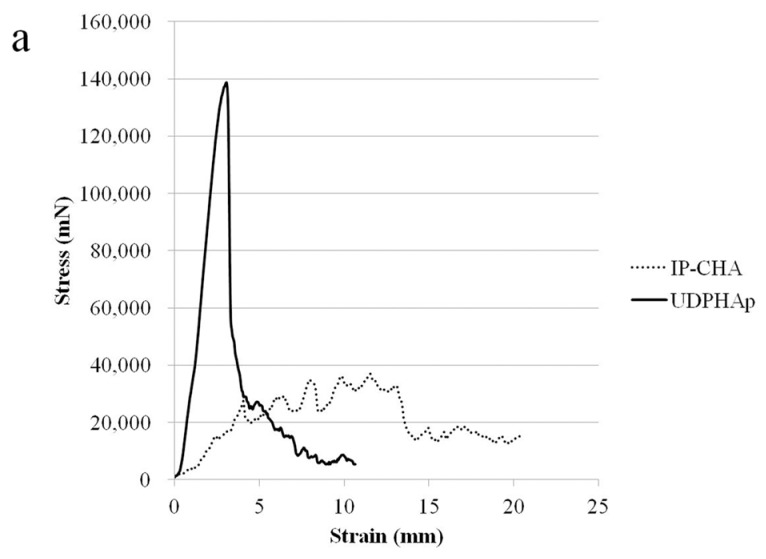

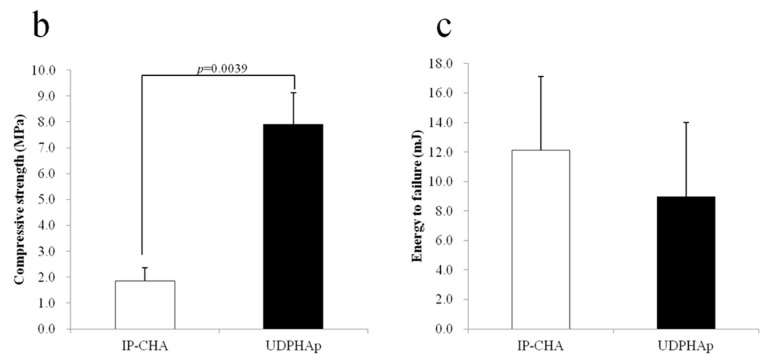
Compression test. (**a**) The representative stress-strain curves of both types of scaffolds; (**b**) compressive strength; and (**c**) energy to failure of both types of scaffolds. Values are mean ± SD; *n* = 6 analyzed by Mann-Whitney U test.

Figure 2a shows the stress-strain curves of the IP-CHA and UDPHAp, respectively. The UDPHAp endured more stress than the IP-CHA at the point of yield but fractures catastrophically, while the IP-CHA showed no clear yield point and exhibits a non-brittle failure by a lower stress than the UDPHAp. This shows that the UDPHAp exhibits a different fracture behavior than that of the IP-CHA. The maximum compressive strengths of the UDPHAp and IP-CHA were 7.89 ± 1.23 MPa and 1.92 ± 0.53 MPa, respectively. The UDPHAp showed a significantly higher maximum compressive strength than the IP-CHA (*p* = 0.0039). However, yield compression after the initial breakdown was longer in the IP-CHA than in the UDPHAp, and breaking energy showed no significant difference between the two groups (8.99 ± 2.72 vs. 13.95 ± 5.69 mJ, *p* = 0.055) (Figure 2b,c).

### 3.3. In Vitro rhBMP-2 Releasing Assay

**Figure 3:**
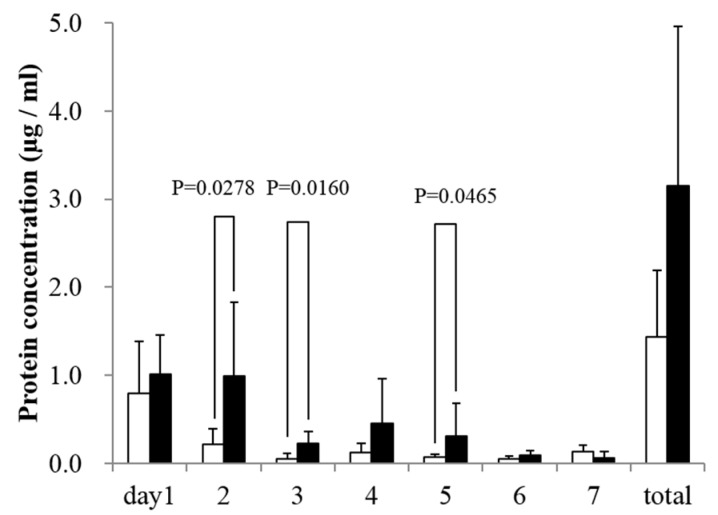
Qualitative rhBMP-2 released from scaffolds. Graph shows protein concentrations of retrieved supernatant of UDPHAp and IP-CHA groups. Mean values were compared using Mann-Whitney U test (*n* = 5).

The rhBMP-2 release was observed in both UDPHAp and IP-CHA groups. On days 2, 3, and 5, UDPHAps released more rhBMP-2 compared to IP-CHAs (*p* = 0.0278, 0.0160, 0.0465, respectively). Total amount of released rhBMP-2 was 3.15 ± 1.81 µg in the UDPHAp group compared to 1.44 ± 0.75 µg in the IP-CHA group as shown in Figure 3.

### 3.4. Observation of Cells Attached to the Scaffolds’ Surface by a Scanning Electron Microscope

**Figure 4:**
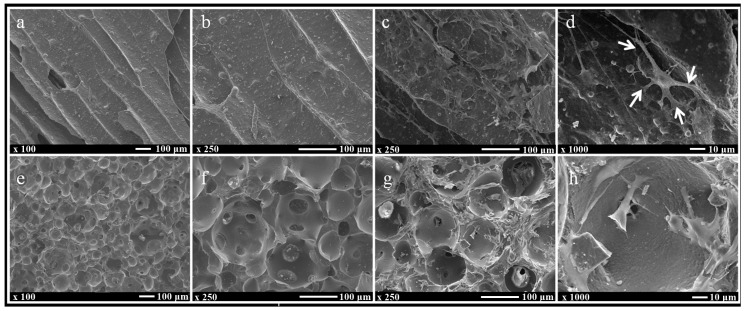
SEM images with and without cells. (**a**,**b**) UDPHAp and (**e**,**f**) IP-CHA without cells; (**c**,**d**) UDPHAp and (**g**,**h**) IP-CHA with MC3T3-E1 cells attached onto each surface of the scaffold. White arrows indicate filopodia. White bars represent 100 µm (**a**–**c** and **e**–**g**) and 10 µm (**d**,**h**).

The UDPHAp had a porous network with a diameter of 100–350 μm, while the pore size of the IP-CHA was 50–250 μm in diameter. The surfaces of the scaffolds were similarly smooth in both the UDPHAp and IP-CHA, and particles of HA were densely arranged (Figure 4a,b,e,f).

Attached cells on the UDPHAp were stuck to the concave surface of the micropore, stretching the filopodia that hooked onto the cells or scaffold’s surface (Figure 4c,d). On the other hand, cells attaching to the IP-CHA surface were rounded and their filopodia were short. There were also some attached cells that blocked the interconnected pores (Figure 4g,h).

### 3.5. Observation of Cell Adhesion by Fluorescence Microscopy

**Figure 5:**
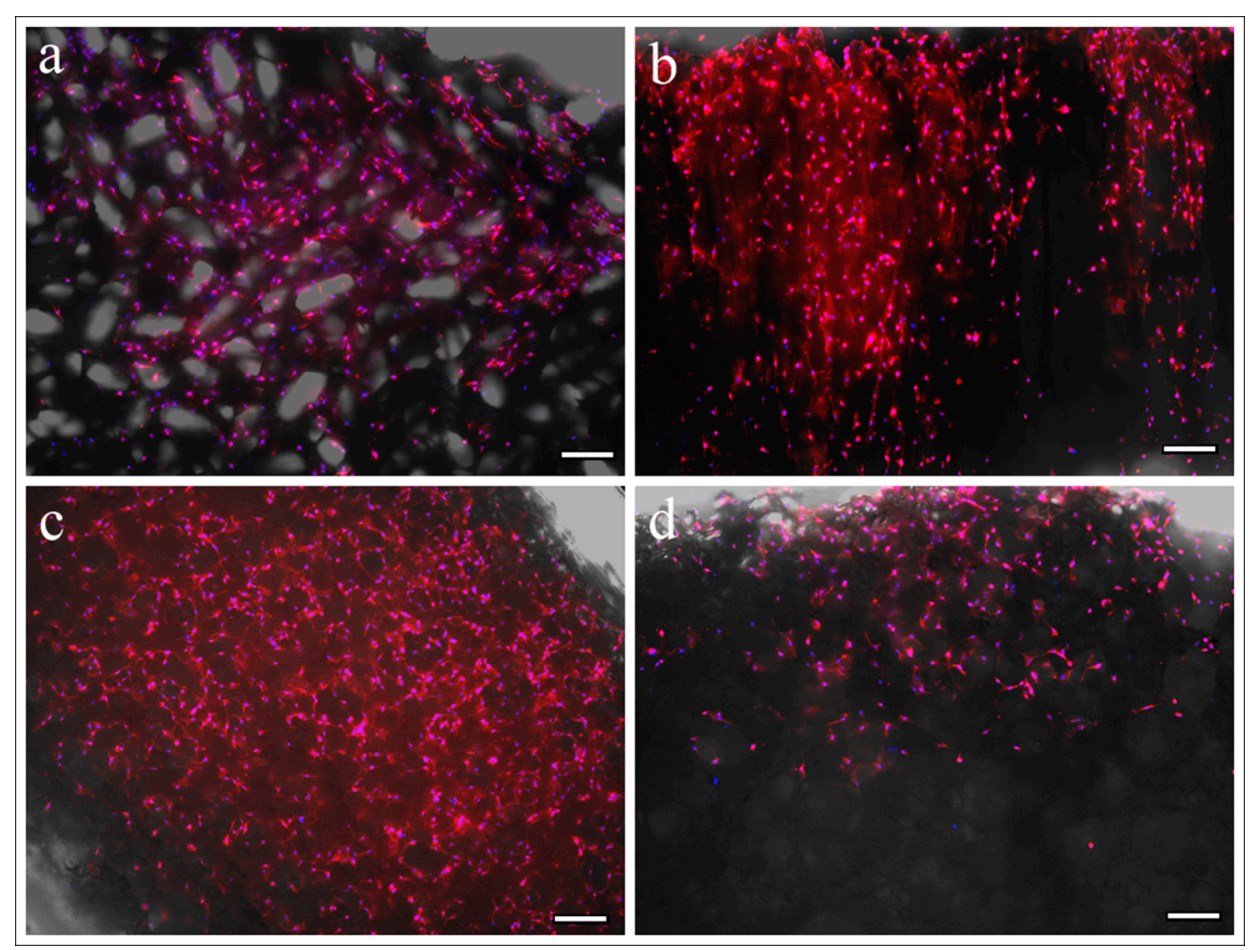
A merge of fluorescent images and bright field images. MC3T3-E1 cells attached onto (**a**,**c**) the upper base and (**b**,**d**) the cross-sectional plane perpendicular to the upper base of (**a**,**b**) UDPHAp and (**c**,**d**) IP-CHA. White bars represent 200 µm.

On the superior aspect of both the UDPHAp and IP-CHA scaffolds, cells were attached to the surface of the scaffolds (Figure 5a,c). Cells on the UDPHAp proliferated more into the internal area of the scaffold than the IP-CHA on cross-sectional observation (Figure 5b,d). 

### 3.6. Seeding Efficiency Analysis

**Figure 6:**
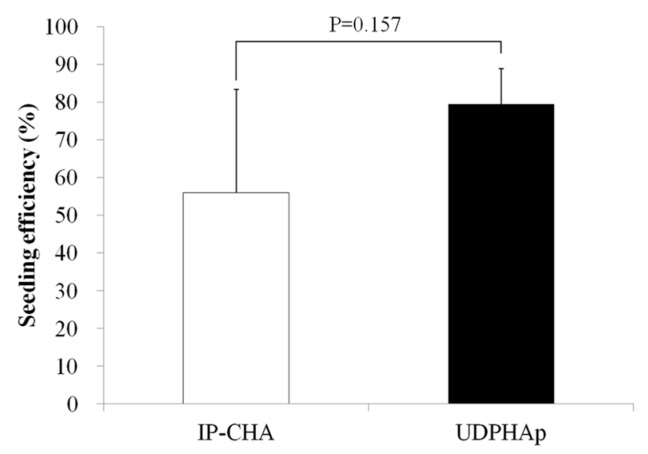
Seeding efficiency for each type of scaffold. Values are mean ± SD; *n* = 5 analyzed by Mann-Whitney U test.

Figure 6 shows the seeding efficiency for each scaffold type. The seeding efficiencies of IP-CHA and UDPHAp were approximately 56% and 79%, respectively. No significant differences were noted in the results between the two scaffolds (*p* = 0.157).

### 3.7. Evaluation of Attached Cell Number

**Figure 7:**
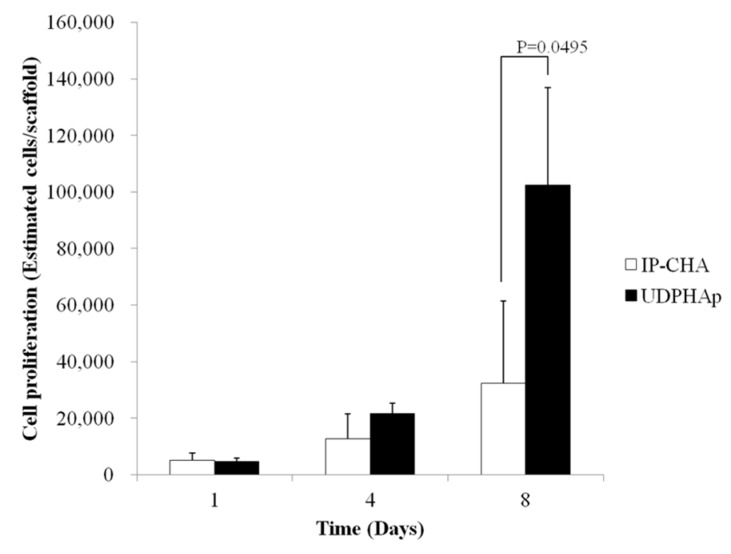
Cell proliferation. Days in culture and estimated number of MC3T3-E1 cells implanted onto IP-CHA and UDPHAp. Values are mean ± SD; *n* = 3 analyzed by Mann-Whitney U test.

On day 1 and day 4, no significant difference in cell proliferation between the UDPHAp and IP-CHA was observed (4627 ± 1213 vs. 5013 ± 2716 cells per scaffold, *p* = 0.5127 and 21,598 ± 3607 vs. 12,570 ± 8792 cells per scaffold, *p* = 0.2752, respectively). On day 8, a significant increase in cell number on the UDPHAp was observed compared to the IP-CHA (102,479 ± 34,391 vs. 32,372 ± 29,061 estimated cells per scaffold, *p* = 0.0495) (Figure 7).

### 3.8. Healing of Mouse Critical-Size Calvarial Bone Defects in Combination with rhBMP-2

**Figure 8:**
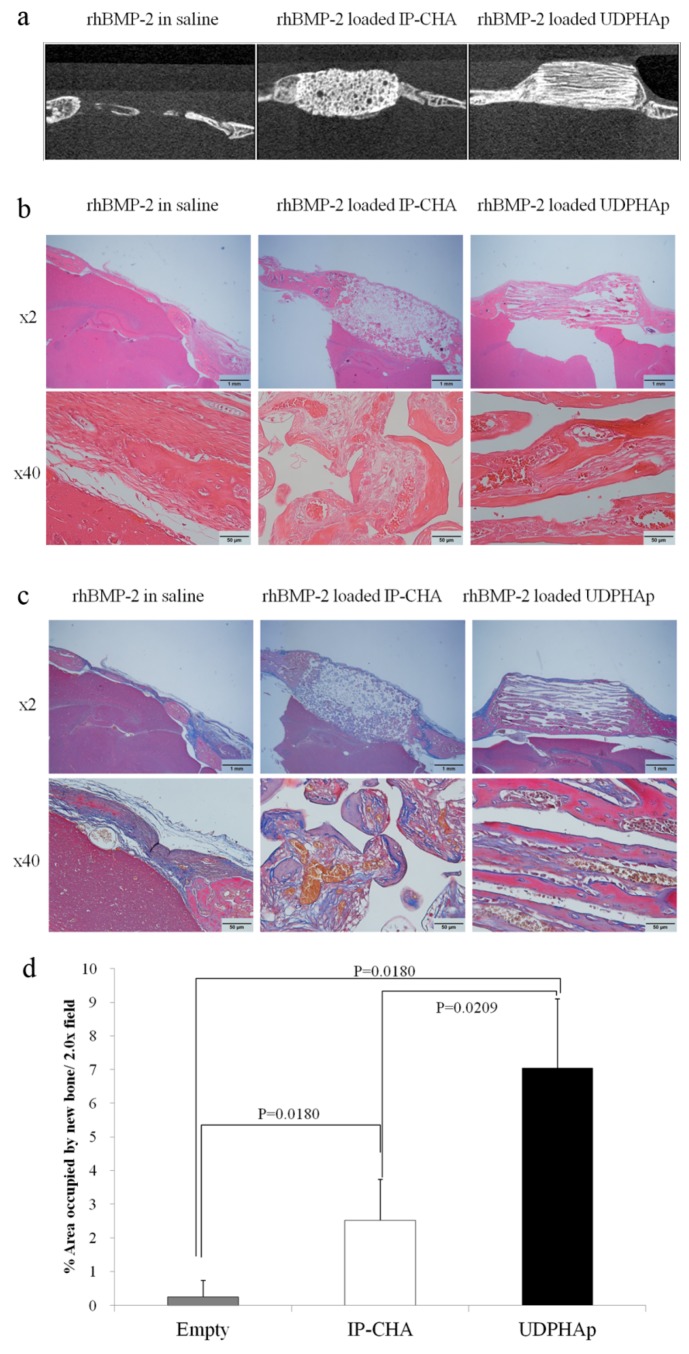
Healing of mouse critical-size calvarial bone defects. Representative images for rhBMP-2 in saline, rhBMP-2 loaded IP-CHA, and rhBMP-2 loaded UDPHAp. (**a**) Representative µCT images (saggital plane) of calvarial defects implanted with saline rhBMP-2, rhBMP-2 loaded IP-CHA and rhBMP-2 loaded UDPHAp three weeks after treatment; Sections were stained with (**b**) Hematoxylin/Eosin (osteoid and nuclei were stained dark pink and dark purple, respectively) and (**c**) Masson’s Trichrome (collagen and woven bone stained blue and mineralized bone stained red); (**d**) Percentage of area occupied by new bone/2.0x field of rhBMP-2 in saline, rhBMP-2 loaded IP-CHA, and rhBMP-2 loaded UDPHAp. Values are mean ± SD; *n* = 4 analyzed by one-way ANOVA and Tukey’s post hoc test.

Micro-CT observation at three weeks after transplantation (Figure 8a) showed more deeply penetrating bone regeneration into the UDPHAp scaffold with rhBMP-2 than the IP-CHA scaffold with rhBMP-2. Little bone regeneration was observed in the Empty group. In histological observation using sections stained with hematoxyline/eosin staining and Masson’s trichrome staining (Figure 8c,d), pores of the IP-CHA group were filled with fibrous tissues (stained blue), whereas those of the UDPHAp group were filled with reproduced bone, which has mature bones (stained red) and woven bones (stained blue). The percentages of new bone area (mature bone + trabecular bone) in the 2x field were 0.243% ± 0.487% for the Empty group, 2.514% ± 1.224% for the IP-CHA group, and 7.045% ± 2.055% for the UDPHAp group. The percentage of new bone area in the UDPHAp group was significantly higher than those of the Empty group and the IP-CHA group (*p* = 0.0180 and *p* = 0.0209, respectively) (Figure 8d).

## 4. Discussion

Design parameters of 3D scaffolds for bone tissue engineering require characteristics such as the biological affinity to make cells efficiently proliferate and differentiate, mechanical properties to reproduce stress transfer, and a suitable pore size. In addition, the ability to hold and gradually release growth factors plays an important role [2,23,24,25]. 

Though the pores of the IP-CHA are spherical, the cellular entry through the communicating channels is tortuous, with a long penetration distance into the interior. In addition, cell invasion may be inhibited in the IP-CHA due to the smaller diameter of the communicating channel than that of each pore. Conversely, because the UDPHAp has a communicating pore structure in the axial direction with no bottlenecks, seeded cells can freely migrate into the given space. Our cell seeding protocol prevents overflow by carefully dropping a small amount of high concentration cell suspension inside the scaffolding material. In the UDPHAp, the cell suspension penetrated quickly into the entire scaffolding material by capillary action. However, in the IP-CHA, droplets of cell suspension that formed on the scaffold surface were not easily absorbed into the scaffold. The cell suspension took about one hour to be entirely absorbed. Cells take approximately 1 h to attach to the scaffold surfaces [26]. If the penetration rate of the cell suspension into the inner region of the scaffold is slow, more cells adhere near the scaffold surface, and less cells enter deeply into the scaffold. Hence, the IP-CHA cells were given little space to grow, and the difference in the attached cell number became greater over time, despite showing no significant difference in cell seeding efficiency.

Despite exhibiting higher porosity than the IP-CHA, the UDPHAp shows stronger compressive strength with respect to the axial direction, which performed at a higher level than the compressive strength of cancellous bone [27]. The strength of scaffolds used in bioengineering with orientation-structured pores is higher than that of random-structured pores with the same porosity [28]. Moreover, characteristics of the UDPHAp are considered especially significant in the treatment of organs such as long bones, in which the orientation plays an important role [29]. Therefore, the UDPHAp is considered an excellent material as a scaffold for bone tissue engineering. 

UDPHAp showed more cell, rhBMP-2, and vascular introduction into the pores. During the fracture repair process, hematoma is formed on the site of the fracture. Growth factors, such as BMPs that are present in hematoma, control the proliferation and differentiation of bone-related cells. In this study, the UDPHAp showed better osteogenic capacity than the IP-CHA with added rhBMP-2. The results may be due to the alignment structure of the UDPHAp, which enabled extracellular fluid to efficiently circulate and growth factors to diffuse uniformly. 5 μL of the BMP solution was not sufficient enough to cover the whole scaffold. UDPHAps can rapidly infiltrate fluids into its scaffolds due to their unidirectional pore structure; therefore, the scaffolds could effectively infiltrate a small amount of BMP solution into deeper sections of its construct. In contrast, convoluted paths are created by randomly interconnected pores of the IP-CHAs, resulting in a considerable number of dead pores. The hindered infiltration from dead pores prevents the BMP solution from reaching deeper depths, especially in small quantities. Because of this morphological difference, the two scaffolds showed a significant difference in the amount of bone regeneration and their distribution.

From the above results, we conclude that the UDPHAp can maintain stress transfer, help in vivo circulation of growth factors, and make bone-related cells efficiently grow and differentiate to facilitate early and reliable bone formation. We believe UDPHAp is not only an excellent 3D scaffold for the treatment of fractures and bone defects, but also for the field of bone tissue engineering, which is expected to play an important part in the future of clinical medicine.
